# The association between elderly people’s sedentary behaviors and their health-related quality of life: focusing on comparing the young-old and the old-old

**DOI:** 10.1186/s12955-019-1191-0

**Published:** 2019-07-26

**Authors:** Yujeong Kim, Eunmi Lee

**Affiliations:** 1 College of Nursing, Kyungpook National University, 680 Gukchabosangro, Jung-gu, Daegu, 41944 Republic of Korea; 2 Department of Nursing, Hoseo University, 20, Hoseo-ro79beon-gil, Baebang-eup, Asan-si, Chungcheongnam-do 31499 Republic of Korea

**Keywords:** Sedentary behaviors, Quality of life, Anxiety, Young-old, Old-old

## Abstract

**Background:**

Research on the effects of sedentary behavior on the health-related quality of life (HRQoL) of the elderly is limited. The present study aimed to determine the association between sedentary behavior and the HRQoL of the young-old (aged 65–74 years) people and old-old (aged ≥75 years) people.

**Methods:**

This study used the raw data of the 7th Korea National Health and Nutrition Examination Survey for 2016. The study subjects were 1,415 people aged over 65 years. The association between HRQoL and average daily sitting time was analyzed using the point biserial correlation coefficient. The effect of sedentary behavior on HRQoL was analyzed by logistic regression analysis.

**Results:**

Overall, elderly people aged ≥65 years spent 7.9 h in sedentary pursuits: the young-old spent 7.7 h and the old-old spent 9.0 h. Longer sitting time was found to be associated with lower HROoL while shorter sitting time was associated with higher HROoL, with the relationship stronger among the old-old than among the young-old. This means that the effects of either having longer time sitting per day and low quality of life or shorter time sitting per day and high quality of life are more pronounced in the old-old in comparison to the young-old.

**Conclusions:**

Sedentary behavior is significantly associated with people’s HRQoL. Interventions towards improving the HRQoL by reducing sedentary behavior based on the respective characteristics of young-old and old-old people are needed.

## Background

Elderly people aged ≥65 years in South Korea constitute 13.8% of the total population, and this share is expected to increase to more than 40% by 2060 [1]. The interest in improving quality of life by improving the health of the elderly and preventing diseases is inevitably increasing because the increase in the elderly population results in increasing social costs. Health-related quality of life (HRQoL) refers to perceived physical and psychosocial health or well-being [2]. Recent evidence suggests that sedentary behavior is inversely associated with HRQoL [3].

Sedentary behavior is defined as any waking behavior characterized by low-energy expenditure while in a sitting or reclining posture [4]. It has been reported that longer durations of sedentary behavior lead to higher obesity, type-2 diabetes, reduced bone density, cardiovascular diseases, and mortality [5–7]. Particularly in the case of elderly people, approximately 60% of their waking hours are spent sitting, and they are the group that has the longest durations of sedentary behavior [8].

A review of the literature on the influence of sedentary behavior on HRQoL indicated that high level of sedentary behavior is significantly related to low level of HRQoL, and some studies [9, 10] have indicated that reducing sedentary behavior significantly improves HRQoL. Research, however, has mostly been conducted on adolescents or adults, and investigation of the effect of sedentary behavior on people’s HRQoL over the age of 65 is limited. In particular, concerns have been raised [11] that studying elderly people with a wide age range (65–95 years of age) may lead to overlooking the differences in health status and problems between the young-old (aged 65–74 years) and the old-old (aged≥75 years). As evidence for the potentially neglected differences, the average healthcare cost of elderly people aged ≥75 years is almost twice as much as that of the young-old, and the old-old are significantly more physically, mentally, and financially vulnerable than the young-old [12]. Accordingly, Negarten, Moorn, and Low [13] proposed classifying the elderly under the age of 75 years as the young-old and those who are 75 years or older as the old-old. As recent studies have found that the average life expectancy has increased to 86 for men and 91 for women in the UK [14], there is a need to evaluate HRQoL, an indicator of the elderly aging process, separately for the young-old and the old-old [15]. Though some studies have verified the association between HRQoL and sedentary behaviors of the elderly [16], few studies have addressed the more refined characteristics of old age by distinguishing between the young-old and the old-old. To close this gap, the present study aimed to examine the association between sedentary behavior and HRQoL by classifying the elderly into the young-old and the old-old to assess whether sedentary behavior affected the two groups differently.

## Methods

### Design

The present study used the raw data obtained in 2016 from the 7th Korea National Health and Nutrition Examination Survey (KNHANES VII) conducted between 2016 and 2018 in the form of a rolling sample survey of people aged ≥ 1 year to investigate the level of health, health behavior, and food and nutrient intake of the South Korean public. The KNHANES is conducted every year to generate basic data for health policies such as setup and evaluation of the goals of the National Health Plan and the development of health promotion programs [17]. The present study analyzed the data of 2016, which was the first year in the 7th survey.

### Study population

A total of 8,150 participants from 3,513 households were surveyed in 2016. Among them, 1,632 participants were 65 years or older. Of the 1,415 participants (excluding missing data) included in this study, 884 were young-old (aged 65–74 years) and 531 were old-old (aged ≥75 years).

The general characteristics of the young-old and the old-old are shown in (Tables 1 and 2). Of the young-old, 47.6% were male and 52.4% were female, 70.4% had low household income and 29.6% had high household income, 70.6% had an education level less than or equal to middle school graduation and 29.4% had an education level greater than or equal to high school graduation, and 76.4% were married. The percentage of participants who had activity limitations was 16.5 and 9.6% were bedridden. Of the old-old, 37.6% were male and 62.4% were female, 79.2% had low household income and 20.8% had high household income, 77.1% had an education level less than or equal to middle school graduation and 22.9% had an education level greater than or equal to high school graduation, and 50.2% were married. The percentage of participants who had activity limitations was 25.3 and 9.5% were bedridden.

### Measures

Sedentary behavior was measured based on the average amount of time spent sitting or lying down per day, elicited by the question “How many hours do you sit or lie down on an average day?” The data obtained by the Korea Centers for Disease Control and Prevention using the EQ-5D-3 L [18] with permission from EuroQol Group were used for the HRQoL data. The EQ-5D-3 L comprises the following five dimensions: mobility, self-care, usual activities, pain/discomfort, and anxiety/depression. Each domain is evaluated on a 3-point scale, and one point was assigned to “no problems”, two points to “some problems”, and three points to “extreme problems.”

### Statistical analysis

The HRQoL (comprising five domains: mobility, self-care, usual activities, pain/discomfort, and anxiety/depression) data according to general characteristics (gender, age, marital status, level of household income, education level, activity limitations, and bedridden) were compared between the two specified elderly groups: the young-old (aged 65–74 years) versus the old-old (aged ≥75 years). To assess the influence of general characteristics on HRQoL, we conducted a Cochran-Mantel-Haenzel test (CMH) with the elderly group as the control variable. The difference in the influence of the elderly group was analyzed by the Brerslow-Day test. The association between sedentary behavior and HRQoL was analyzed according to the presence or absence of control variables. First, the point biserial correlation coefficient was calculated to assess the association without control variables given that the five domains of HRQoL are all binary qualitative variables. Although the point biserial correlation coefficient confirmed the Pearson’s correlation coefficient, it cannot be used for a hypothesis test. Therefore, the Pearson’s correlation coefficient was calculated for the hypothesis test. Second, holding general characteristics as control variables, the association between sedentary behavior and HRQoL was analyzed by logistic regression analysis, with HRQoL as a dependent variable and the average daily sitting time as an independent variable. Gender, household income level, education level, marital status, activity limitations, and being bedridden were taken as control variables.

## Results

On average, participants spent 7.9 h/day in sedentary behaviors. Young-old spent 7.7 h/day on average while the old-old spent 9.0 h/day on average.

### Differences in the quality of life according to general characteristics

Among the areas of quality of life, results indicated significant differences in mobility, self-care, usual activities, and pain/discomfort and no significant differences in anxiety/depression between the two elderly groups. Mobility (CMH 34.83, *p* < 0.001), self-care (CMH 12.26, *p* = 0.001), usual activities (CMH 31.33, *p* < 0.001), and pain/discomfort (CMH 6.70, *p* = 0.010) were all found more problematic for the old-old in comparison to the young-old (Tables 1 and 2).

**Table 1:** HRQoL of young-old and old-old elderly according to general characteristics I

		general characteristics			Mobility					Self-care				
		total	young-old	old-old	total	young-old	old-old	CMH *x*^2^(p)	Breslow-Day *x*^2^(p)	total	young-old	old-old	CMH *x*^2^(p)	Breslow-Day *x*^2^(p)
Total			60.4	39.6	37.8	30.6	48.6	34.831 (< 0.001)		11.5	8.8	15.7	12.258 (0.001)	
Gender	Male	43.6	47.6	37.6	26.8	21.0	38.1	48.063 (< 0.001)	0.726 (0.394)	10.5	8.1	15.3	0.431 (0.512)	0.125 (0.724)
	Female	56.4	52.4	62.4	46.2	39.3	55.0			12.3	9.5	15.9		
Household income	Low	73.9	70.4	79.2	41.1	34.4	50.2	11.081 (0.001)	1.701 (0.192)	13.6	10.6	17.7	12.925 (< 0.001)	0.000 (0.993)
	High	26.1	29.6	20.8	29.5	22.6	44.5			6.0	4.9	8.5		
Education level	Middle school or less	73.2	70.6	77.1	42.6	35.3	52.7	34.618 (< 0.001)	0.151 (0.698)	12.8	10.1	16.5	5.622 (0.018)	0.197 (0.657)
	High school or more	26.8	29.4	22.9	24.3	19.0	34.8			7.8	5.8	11.7		
Marital status	Married	66.0	76.4	50.2	32.9	27.7	45.1	12.755 (< 0.001)	1.342 (0.247)	10.1	7.9	15.4	1.612 (0.204)	1419 (0.234)
	Single	34.0	23.6	49.8	47.2	40.2	52.3			14.3	11.9	16.0		
Activity limits	Yes	19.9	16.5	25.3	68.0	62.6	73.3	123.561 (< 0.001)	0.755 (0.385)	29.0	22.6	35.4	95.064 (< 0.001)	0.347 (0.556)
	No	80.1	83.5	74.7	30.3	24.3	40.4			7.2	6.1	9.0		
Bed-Ridden	Yes	9.6	9.6	9.5	68.0	60.6	79.3	60.608 (< 0.001)	0.082 (0.775)	29.6	21.6	42.1	49.168 (< 0.001)	0.654 (0.419)
	No	90.4	90.4	90.5	34.5	27.3	45.4			9.6	7.4	12.9

**Table 2:** HRQoL of young-old and old-old elderly according to general characteristics II

		Usual activities					Pain/discomfort					Anxiety/depression				
		total	young-old	old-old	CMH *x*^2^(p)	Breslow-Day *x*^2^(p)	total	young-old	old-old	CMH *x*^2^(p)	Breslow-Day *x*^2^(p)	total	young-old	old-old	CMH *x*^2^(p)	Breslow-Day *x*^2^(p)
Total		21.7	15.9	30.7	31.330 (< 0.001)		35.2	32.2	39.8	6.695 (0.010)		15.4	14.5	16.8	1.346 (0.246)	
Gender	Male	14.6	10.1	23.1	27.266 (< 0.001)	0.894 (0.345)	22.7	17.9	32.0	71.076 (< 0.001)	10.806 (0.001)	12.0	9.2	17.3	9.468 (0.002)	8.579 (0.003)
	Female	27.3	21.1	35.2			44.9	45.2	44.5			18.1	19.4	16.5		
Household income	Low	24.9	19.1	32.9	18.355 (< 0.001)	2.091 (0.148)	38.2	36.3	40.7	11.150 (0.001)	3.676 (0.055)	18.0	17.7	18.5	20.885 (< 0.001)	0.544 (0.461)
	High	12.9	8.1	23.3			27.7	23.0	37.9			7.8	6.9	9.8		
Education level	Middle school or less	24.6	18.0	33.8	16.065 (< 0.001)	0.005 (0.946)	39.3	37.2	42.3	27.487 (< 0.001)	2.513 (0.113)	16.8	15.9	17.9	6.008 (0.014)	0.030 (0.863)
	High school or more	13.7	10.0	20.9			23.7	19.5	31.9			11.3	11.0	11.8		
Marital status	Married	17.3	13.0	27.3	15.366 (< 0.001)	3.213 (0.073)	31.1	27.7	39.0	14.982 (< 0.001)	10.140 (0.002)	13.1	10.7	18.6	10.405 (0.001)	21.639 (< 0.001)
	Single	30.2	25.0	34.0			43.2	46.8	40.7			20.0	27.1	14.9		
Activity limits	Yes	51.7	46.6	56.8	167.526 (< 0.001)	3.352 (0.067)	67.0	66.0	68.0	147.895 (< 0.001)	0.269 (0.604)	33.1	36.3	29.8	81.146 (< 0.001)	2.657 (0.103)
	No	14.3	9.8	21.9			27.4	25.6	30.5			11.1	10.3	12.5		
Bed-Ridden	Yes	46.6	39.0	58.2	56.730 (< 0.001)	0.134 (0.715)	69.8	72.8	65.2	79.214 (< 0.001)	3.892 (0.049)	40.3	45.5	32.3	71.396 (< 0.001)	5.218 (0.022)
	No	19.0	13.3	27.8			31.5	27.8	37.1			12.7	11.1	15.2		

In order to identify the impact of general characteristics, differences in quality of life due to general characteristics were controlled for and explored. The results showed significant differences in gender, age, household income level, education level, marital status, activity limitations, and being bedridden, except that marital status had no effect on the quality of life area of self-care. Specifically, women in comparison to men, those with low household income compared to those with high household income, those with low level of education in comparison to those with high level of education, those without a spouse in comparison to those with, those who are limited in activities in comparison to those who are not, and those with an illness in comparison to those without were shown to have more problems in all areas of quality of life.

Investigating the differences in general characteristics between the young-old and the old-old, we found no significant differences in mobility, self-care, and usual activities and significant differences in pain/discomfort and anxiety/depression. We found that the difference in quality of life relating to pain/discomfort due to gender (CMH 71.08, B-D 10.81, *p* = 0.001) and being bedridden (CMH 79.21, B-D 3.89, *p* = 0.049) was greater in the young-old than in the old-old, and that pain/discomfort due to marital status was statistically significant in the young-old but not in the old-old. No statistically significant differences were found between the young-old and the old-old in pain/discomfort due to household income, education level, and activity limitations. In the anxiety/depression area, the difference in the quality of life relating to anxiety/depression due to being bedridden was greater in the young-old than in the old-old (CMH 71.40, B-D 5.22, *p* = 0.022), and that anxiety/depression due to gender and marital status was statistically significant in the young-old but not in the old-old. No statistically significant differences were found between the young-old and the old-old in terms of pain/discomfort due to household income, education level, and activity limitations.

### The association between time spent on sedentary behavior and HRQoL

A significant relationship was found between the quality of life and the average daily sitting time in elderly people (Table 3). Average sedentary time was positively correlated with the five areas of quality of life, suggesting that elderly people who spend more time sitting daily tend to have problems in the areas of quality of life (mobility, self-care, usual activities, pain/discomfort, and anxiety/depression *p* < 0.001), and conversely, those who spend less time sitting daily tend not to have these problems. In other words, elderly people either have longer daily sitting time and low quality of life, or shorter daily sitting time and high quality of life. The relationship is stronger among the old-old than among the young-old. This means that the characteristics of either having longer time sitting per day and low quality of life or shorter time sitting per day and high quality of life are more pronounced in the old-old in comparison to the young-old.

**Table 3:** Association between sedentary behaviors and HRQoL

	Total		Young-old		Old-old	
	*r*_*pb*_	*p*-value	*r*_*pb*_	*p*-value	*r*_*pb*_	*p*-value
Mobility	0.214	< 0.001	0.139	0.004	0.251	< 0.001
Self-care	0.175	< 0.001	0.148	0.001	0.173	0.001
Usual activities	0.257	< 0.001	0.205	< 0.001	0.264	< 0.001
Pain/discomfort	0.166	< 0.001	0.127	0.002	0.192	< 0.001
Anxiety/depression	0.171	< 0.001	0.097	0.032	0.260	< 0.001

*r*_*pb*_ is a point biserial correlation coefficient

### The influence of time spent on sedentary behavior on HRQoL

Results revealed a statistically significant effect of average daily sitting time on the HRQoL dimensions (Table 4). For all participants, a one-hour increase in sedentary time led to 1.089 times higher odds of mobility problems (95% CIs 1.04–1.14, *p* < 0.001), 1.117 times higher odds of self-care problems (95% CIs 1.06–1.18, *p* < 0.001), 1.145 times higher odds of usual activities problems (95% CIs 1.09–1.20, *p* < 0.001), 1.059 times higher odds of pain/discomfort problems (95% CIs 1.02–1.10, *p =* 0.004), and 1.100 times higher odds of anxiety/depression (95% CIs 1.04–1.16, *p =* 0.001).

**Table 4:** Effects of sedentary behaviors on HRQoL

	Total		Young-old		Old-old	
	OR (95% CI)	wald, t (p)	OR (95% CI)	wald, t (p)	OR (95% CI)	wald, t (p)
Mobility	1.089 (1.042, 1.137)	14.663 (< 0.001)	1.054 (0.987, 1.124)	2.486 (0.115)	1.130 (1.064, 1.199)	16.116 (< 0.001)
Self-care	1.117 (1.056, 1.183)	14.705 (< 0.001)	1.120 (1.025, 1.223)	6.274 (0.012)	1.110 (1.027, 1.201)	6.896 (0.009)
Usual activities	1.145 (1.091, 1.202)	29.947 (< 0.001)	1.138 (1.064, 1.216)	14.289 (< 0.001)	1.156 (1.082, 1.237)	18.106 (< 0.001)
Pain/discomfort	1.059 (1.019, 1.100)	8.369 (0.004)	1.035 (0.977, 1.096)	1.344 (0.246)	1.093 (1.03, 1.16)	8.732 (0.003)
Anxiety/depression	1.100 (1.043, 1.159)	12.347 (0.001)	1.025 (0.954, 1.101)	0.455 (0.500)	1.224 (1.127, 1.328)	23.242 (< 0.001)

^a^ Control variables: gender, household income, education level, marital status, activity limits, bed-ridden

In the case of the young-old, the effects were statistically nonsignificant in the dimensions of mobility, pain/discomfort, and anxiety/depression. When sedentary time increased by one hour, however, the odds of self-care problems and usual activities problems were 1.120 times (95% CIs 1.03–1.22, *p =* 0.012) and 1.138 times higher (95% CIs 1.06–1.22, *p* < 0.001), respectively.

In the case of the old-old, when sedentary time increased by one hour, the odds were 1.130 times higher of mobility problems (95% CIs 1.06-1.20, *p* <0.001), 1.110 times higher of self-care problems (95% CIs 1.03–1.20, *p =* 0.009), 1.156 times higher of usual activities problems (95% CIs 1.08–1.24, *p* < 0.001), 1.093 times higher of pain/discomfort problems (95% CIs 1.03–1.16, *p =* 0.003), and 1.224 times higher of anxiety/depression (95% CIs 1.13–1.33, *p* < 0.001).

## Discussion

The major findings of the study can be summarized as follows. First, overall, participants spent 7.9 h/day in sedentary behaviors on average, with 7.7 h/day in the young-old, and 9.0 h/day in the old-old. This is longer compared with the reported average sitting time of 5.8 h/day in adults aged 18–65 years in 20 countries [19]. Although it seems somewhat shorter than the reported 8.5 h/day in elderly people in the U.S. [20], it is longer than 6.0 h/day in England [21] and 7.4 h/day in Spain [22]. One possible reason for the Korean elderly’s longer sitting time in comparison to their international peers is thought to be that more than 90% of the Korean elderly spend most of their leisure time watching TV or playing Korean games such as hawtu, baduk, and janggi, which are played sitting down at senior centers [23]. However, these sedentary social and cognitive activities are also related to the wellness of the elderly and low risk for dementia [24]. Therefore, future studies should not only consider the length of time spent sitting but also the social and cognitive activities performed during those sedentary periods. In addition, sedentary time of the elderly may differ according to country, with the possible influences of culture or ethnicity on the overall life habits of the elderly including sedentary behavior; however, cross-country analysis on the topic is rare. Some research has revealed the influence of environmental factors such as residential areas [25] including rural areas and cities, welfare facilities for the elderly [26], resting places [27], and housing structures [26] on sitting time of the elderly. Accordingly, further research needs to be conducted on environmental and cultural factors that influence the sedentary behavior of the elderly.

Second, the present study found that sedentary time is longer among the old-old than the young-old. This finding is consistent with previous research [8, 16], and Shiroma, Freedson, Trost, and Lee [28] also reported that as age increased by 1 year for elderly aged ≥65 years, total daily sedentary time per year increased by approximately 5%. This is due to the increased mobility impairment as aging progresses from young-old to old-old [25]. Compared to the young-old who are comparatively healthy, active, and independent, the old-old spend a long time sitting due to the fact that they are the age group to be most directly affected by mobility disorders resulting from chronic health issues such as arthritis and pain [29]. Therefore, interventions to minimize the number of chronic illnesses in the old-old who have mobility disorders are needed to ensure that the elderly people are healthy and able to maintain independent body functions. In addition, consistent management and implementation are necessary in promoting preventative programs that can prevent the development of diseases among the elderly people with chronic diseases.

Third, there were significant differences only in the pain/discomfort and anxiety/depression areas of quality of life between the effect of general characteristics on HRQoL in the young-old and the old-old. The young-old people had larger differences in quality of life relating to pain/discomfort based on gender and whether or not they were bedridden in comparison to the old-old. Additionally, there were significant differences in pain/discomfort and anxiety/depression due to marital status only in the young-old. Because the young-old often have jobs, pain/discomfort problems can have a bigger impact on the quality of life relating to financial activities and health [30]. In addition, the young-old have a higher chance to receive help, share emotions, allow financial help, and interact socially through their spouse which can have a positive impact on HRQoL such as lessening the anxiety, depression, and discomfort felt [31]. The reason that there were no significant differences in HRQoL relating to other general characteristics in the old-old could be that the old-old tend to find meaning in existence itself and adapt to and find joy in the current situation despite cognitive and functional limitations, financial situation, and isolation from society and family [32]. However, the research results on the quality of life differences due to general characteristics such as gender, financial situation, and existence of spouse between the young-old and the old-old are not consistent. Therefore, a more definitive study in the future is needed to identify the changes and affecting factors.

Fourth, the results of the current study show that daily sitting time in the elderly and the five sub-areas of HRQoL had a significant positive correlation. This is similar to other related studies on elderly people [16]. In particular, the current study found that the association between sedentary activity and HRQoL was stronger in the young-old than in the old-old. Also, in comparison to the young-old, the HRQoL areas of usual activities, anxiety/depression, and mobility were highly related to sedentary activity in the old-old. The reason behind these results may be that the old-old experience decreased mobility and increased cognitive problems such as dementia due to chronic illnesses, which may pose problems with usual activities and thus increase sedentary activities [32]. The results can also be explained by the fact that the decrease in mobility and cognitive functions have a direct effect on sedentary activities and self-efficacy, and self-efficacy in turn can exacerbate anxiety and depression and affect the psychological quality of life [33]. In addition, TV or computer usage which comprise a large portion of sedentary activities can hinder social relationships including reducing family time and thus can negatively affect the quality of life by increasing anxiety and depression [34]. Accordingly, in planning healthcare services for elderly with mobility difficulties, services meant to reduce sedentary behavior and maintain physical activities should be developed with consideration of the level of activities. In addition, sedentary time should be integrated into movement behavior guidelines for the elderly, who would benefit from sitting less, breaking up their sitting time, and moving more.

Lastly, comparisons between the young-old and the old-old indicated that the latter have higher odds of problems in all five dimensions of HRQoL with increase in sedentary time. On the other hand, the young-old had higher odds of self-care and usual activities problems, but no statistical significant influence on mobility, pain/discomfort, and anxiety/depression. The reason why sedentary time had no impact on mobility, pain/discomfort, and anxiety/depression of the young-old but affected the old-old may be that the young-old are relatively healthy, active, and living independently [35]. According to a study by Choi [36], only 7% of South Korean elderly aged between 65 and 74 need assistance in personal daily activities, but the proportion rapidly increases after age 75 years, with 40% or more of the old-old aged ≥85 years reported to be in a dependent state. Furthermore, factors such as chronic diseases, deterioration of financial situation, isolation from society and family, and cognitive impairment have been reported to cause depression with increase in age [32]. That is, the old-old are more prone to anxiety and depression due to increased sedentary behavior caused by activity limitations, which are aggravated by socioeconomic responsibilities, loss of independent functioning in everyday life, and alienation from family. Therefore, the old-old would have more accessibility to services if community-based health promotion programs offered activities to reduce sedentary behavior. This would be beneficial not only because of the old-old elderly’s lower education and socioeconomic levels compared to the young-old, but also due to their decrease in physical functioning. Accordingly, when visiting healthcare services are provided for the old-old with consideration of cohabiting family, services meant to reduce sedentary behavior and promote physical activities should be developed with consideration of the level of activities.

## Conclusions

The results of the current research have revealed associations between sedentary activity and health-related areas of quality of life as the elderly get older. Therefore, it is necessary to identify influencing factors of the sedentary behavior of the elderly, develop intervention programs to reduce sedentary behavior, and strengthen welfare systems and policies for the elderly within the community. Research to confirm the effectiveness of these interventions and strategies is also needed. The present study is limited in that the causality among variables cannot be determined due to its cross-sectional design. In addition, sedentary behavior was measured by a self-reported questionnaire, rather than accurate measurements. Future research can obtain objective measurements using instruments such as accelerometers and compare results with the self-reported survey results. Longitudinal research is also needed to further investigate the health-related outcomes including quality of life measures by evaluating the length and social and cognitive aspects of each sedentary activity type. Furthermore, research is called for to identify the environmental and cultural factors that may affect the sedentary behavior of the elderly and to monitor causality of sedentary behavior and life quality for an extended period. Lastly, ongoing interventional studies are needed to improve health-related quality of life in relation to the sedentary behavior of the elderly.

## Data Availability

The datasets used during the current study are available from the corresponding author on reasonable request.

## Abbreviations

HRQoL: Health-related quality of life
KNHANES: Korea National Health and Nutrition Examination Survey
